# Kynurenic Acid and Its Analogue SZR-72 Ameliorate the Severity of Experimental Acute Necrotizing Pancreatitis

**DOI:** 10.3389/fimmu.2021.702764

**Published:** 2021-10-21

**Authors:** Zsolt Balla, Eszter Sára Kormányos, Balázs Kui, Emese Réka Bálint, Gabriella Fűr, Erik Márk Orján, Béla Iványi, László Vécsei, Ferenc Fülöp, Gabriella Varga, András Harazin, Vilmos Tubak, Mária A. Deli, Csaba Papp, Attila Gácser, Tamara Madácsy, Viktória Venglovecz, József Maléth, Péter Hegyi, Lóránd Kiss, Zoltán Rakonczay

**Affiliations:** ^1^ Department of Pathophysiology, University of Szeged, Szeged, Hungary; ^2^ Department of Medicine, University of Szeged, Szeged, Hungary; ^3^ Department of Pathology, University of Szeged, Szeged, Hungary; ^4^ Department of Neurology, Interdisciplinary Excellence Centre, University of Szeged, Szeged, Hungary; ^5^ Hungarian Academy of Sciences-University of Szeged Neuroscience Research Group, Hungarian Academy of Sciences – University of Szeged, Szeged, Hungary; ^6^ Institute of Pharmaceutical Chemistry, University of Szeged, Szeged, Hungary; ^7^ Stereochemistry Research Team, Hungarian Academy of Sciences – University of Szeged, Szeged, Hungary; ^8^ Institute of Surgical Research, University of Szeged, Szeged, Hungary; ^9^ Institute of Biophysics, Biological Research Centre, Szeged, Hungary; ^10^ Creative Laboratory Ltd., Szeged, Hungary; ^11^ Department of Microbiology, University of Szeged, Szeged, Hungary; ^12^ Hungarian Academy of Sciences-University of Szeged Lendület Mycobiome Research Group, University of Szeged, Szeged, Hungary; ^13^ Department of Pharmacology and Pharmacotherapy, University of Szeged, Szeged, Hungary; ^14^ Hungarian Academy of Sciences-University of Szeged Translational Gastroenterology Research Group, Szeged, Hungary; ^15^ Institute for Translational Medicine, University of Pécs, Pécs, Hungary

**Keywords:** acute pancreatitis, kynurenic acid, SZR-72, NMDA receptor-1, NMDA, tryptophan pathway, N-methyl-D-aspartate

## Abstract

The pathophysiology of acute pancreatitis (AP) is not well understood, and the disease does not have specific therapy. Tryptophan metabolite L-kynurenic acid (KYNA) and its synthetic analogue SZR-72 are antagonists of the N-methyl-D-aspartate receptor (NMDAR) and have immune modulatory roles in several inflammatory diseases. Our aims were to investigate the effects of KYNA and SZR-72 on experimental AP and to reveal their possible mode of action. AP was induced by intraperitoneal (i.p.) injection of L-ornithine-HCl (LO) in SPRD rats. Animals were pretreated with 75-300 mg/kg KYNA or SZR-72. Control animals were injected with physiological saline instead of LO, KYNA and/or SZR-72. Laboratory and histological parameters, as well as pancreatic and systemic circulation were measured to evaluate AP severity. Pancreatic heat shock protein-72 and IL-1β were measured by western blot and ELISA, respectively. Pancreatic expression of NMDAR1 was investigated by RT-PCR and immunohistochemistry. Viability of isolated pancreatic acinar cells in response to LO, KYNA, SZR-72 and/or NMDA administration was assessed by propidium-iodide assay. The effects of LO and/or SZR-72 on neutrophil granulocyte function was also studied. Almost all investigated laboratory and histological parameters of AP were significantly reduced by administration of 300 mg/kg KYNA or SZR-72, whereas the 150 mg/kg or 75 mg/kg doses were less or not effective, respectively. The decreased pancreatic microcirculation was also improved in the AP groups treated with 300 mg/kg KYNA or SZR-72. Interestingly, pancreatic heat shock protein-72 expression was significantly increased by administration of SZR-72, KYNA and/or LO. mRNA and protein expression of NMDAR1 was detected in pancreatic tissue. LO treatment caused acinar cell toxicity which was reversed by 250 µM KYNA or SZR-72. Treatment of acini with NMDA (25, 250, 2000 µM) did not influence the effects of KYNA or SZR-72. Moreover, SZR-72 reduced LO-induced H_2_O_2_ production of neutrophil granulocytes. KYNA and SZR-72 have dose-dependent protective effects on LO-induced AP or acinar toxicity which seem to be independent of pancreatic NMDA receptors. Furthermore, SZR-72 treatment suppressed AP-induced activation of neutrophil granulocytes. This study suggests that administration of KYNA and its derivative could be beneficial in AP.

## Introduction

Acute pancreatitis (AP) is a relatively common disease among gastrointestinal disorders (1) with increasing incidence over time (2). Its overall mortality is about 2%, but in severe cases this can reach 30% (3). AP may appear in mild, moderately severe or severe forms based on the Revised Atlanta Classification (4). The pathomechanism of AP is complex and not fully understood. It involves toxic cellular Ca^2+^ overload causing nuclear factor-κB activation in pancreatic acinar cells, impaired autophagy, mitochondrial dysfunction, release of reactive oxygen species (ROS), as well as premature activation of digestive enzymes like trypsinogen (5–8). These events lead to release of tumor necrosis factor α (TNF-α), cytokines (e.g. interleukin 1) and chemokines, which participate in leukocyte recruitment. Neutrophils are the first immune cells reaching pancreatic parenchyma. These cells also activate trypsinogen in acinar cells (9), release inflammatory cytokines or chemokines, secrete myeloperoxidase and reactive oxygen species e.g. hydrogen peroxide (H_2_O_2_), which all contribute to further aggravation of AP (10). Unfortunately, AP management is still based on supportive therapy without specific drugs available.

**Figure 1:**
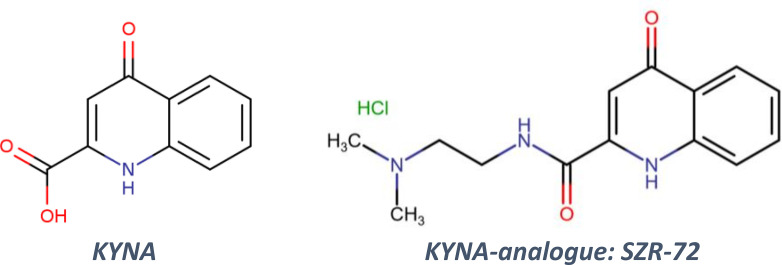
The structure of kynurenic acid (KYNA) and its analogue SZR-72 (2-(2-*N,N*-dimethylaminoethylamine-1-carbonyl)-1*H*-quinolin-4-one hydrochloride).

Tryptophan and its metabolites are important participants of cellular processes, especially in neuronal cells. L-tryptophan is metabolized to N-formyl-L-kynurenine and L-kynurenine (KYN). KYN is further converted to kynurenic acid (KYNA, **Figure 1**), 3-hydroxy-L-kynurenine (3-HK), or anthranilic acid depending on the enzymes (11). Metabolites of the tryptophan-KYN pathway have several effects on both innate and adaptive immune responses (12). KYNA acts as an antagonist on N-methyl-D-aspartate receptor (NMDAR) and has neuroprotective effects (11). It also reduces ischemia/reperfusion-induced retinal ganglion cell death (13). Recently, SZR-72, a promising derivative of KYNA, was also investigated by different research groups (**Figure 1**). SZR-72 readily crosses the blood-brain barrier but KYNA is poorly permeable (14, 15). SZR-72 effectively modulated mitochondrial respiration, while KYNA could restore microcirculation in sepsis (16). KYNA and SZR-72 suppressed pro-inflammatory factors released by mononuclear cells and neutrophils e.g. TNF-α, high mobility group box protein 1, and human neutrophil peptide 1–3 (17). Furthermore, in that study SZR-72 showed more potent effects than KYNA. SZR-72 could also suppress inflammation in the colon through antagonism of NMDAR (18).

AP severity was shown to be influenced by metabolites of the tryptophan pathway or by disturbance of this pathway. Overall, 3-HK generates free radicals and causes cytotoxicity, while KYNA inhibits inflammation, prevents lipid peroxidation and ROS generation (19). 3-HK concentration is increased during AP in human samples and its plasma levels correlated with the progression of systemic inflammation and the severity of AP (20). The inhibitors of kynurenine-3-monoxigenase reduce the production of 3-HK. Application of such an inhibitor prevented multiple organ failure in experimental AP in rodents (21). In contrast to 3-HK, the effect of endogenous KYNA or its synthetic derivate SZR-72 is unknown during AP.

Based on the previously detected promising anti-inflammatory roles of KYNA and SZR-72, our aim was to investigate the effects of these molecules on the severity of experimental AP. Furthermore, we wanted to reveal whether they act *via* NMDAR.

## Materials and Methods

### Materials

All chemicals were purchased from Sigma-Aldrich (Budapest, Hungary) unless indicated otherwise. SZR-72 [2-(2-N,N-dimethylaminoethyl-amine-1carbonyl)-1H-quinolin-4-one hydrochloride] was synthesized by the Institute of Pharmaceutical Chemistry (University of Szeged, Hungary).

The solutions used for *in vivo* measurements were freshly prepared before each experiment. L−ornithine-HCl (LO, 300 mg/ml), kynurenic acid (KYNA, 50 mg/ml), and SZR−72 (50 mg/ml) were dissolved in physiological saline (PS) and the pH of the solutions was adjusted to 7.35-7.4 (KYNA and SZR-72 precipitate above pH 7.4).

### Animals

Male Sprague-Dawley (SPRD) rats weighing 200-250 g were used for the experiments. The animals were kept at a constant room temperature of 23°C with a 12-hour light–dark cycle and were allowed free access to water and standard laboratory chow for rodents (Biofarm, Zagyvaszántó, Hungary). Our experiments were executed according to the European Union Directive 2010/63/EU and the Hungarian Government Decree 40/2013 (II.14.). Experiments were approved by both local (University of Szeged) and national ethics committees (X/3353/2017.) for investigations involving animals.

### *In Vivo* Experiments: Acute Pancreatitis Induction, Treatment With Kynurenic Acid or SZR−72, and Tissue Harvesting

Necrotizing AP was induced by intraperitoneal (i.p.) injection of 3 g/kg LO administered in the morning. A single i.p. injection of kynurenic acid (KYNA) or SZR-72 (75, 150, or 300 mg/kg) was administered 1 hour prior to the induction of AP. Control animals were treated with physiological saline (PS) instead of LO, KYNA and/or SZR-72. Animals were sacrificed 24 h after the LO injection (at the peak of pancreatic inflammation) by deep anesthesia with 45 mg/kg i.p. pentobarbital (Bimeda MTC, Cambridge, Canada). Blood was collected *via* cardiac puncture, then the pancreas was rapidly removed. Pancreatic tissue was cleaned from fat and lymph nodes on ice, then cut into pieces. One large piece was immediately frozen in liquid nitrogen and stored at –80°C until biochemical assays were performed. Another piece of the pancreas was fixed in 8% neutral formaldehyde solution for histological analysis. The third piece was stored in Eppendorf tubes at room temperature for dry-wet weight measurement. The last piece was frozen in cryomatrix for sectioning and immunofluorescent stainings. Blood samples were centrifuged at 2500 RCF for 15 mins at 4°C, sera were collected and stored at –20°C until use.

After LO administration, animals showed signs of sickness and became sluggish as expected. However, a few of them got depressed and lethargic within 12 h after the LO injection. The core temperature of these animals was monitored with a rectal digital thermometer. Once it decreased to a critical level (27-29°C), rats were euthanized by pentobarbital overdose (200 mg/kg i.p.) to minimize suffering. The percentage of euthanized rats was 3% in the LO treated groups. Surviving animals either developed necrotizing AP or remained AP−free by the time the experiment was terminated (24 h).

### Histological Analysis

Formalin-fixed pancreatic tissues were sectioned to 3 µm. These sections were prepared and stained with hematoxylin and eosin and were analyzed and scored by a pathologist blinded to the experimental protocol (22). Edema was scored from 0−3 points (0: none; 1: patchy interlobular; 2: diffuse interlobular; 3: diffuse interlobular and intra-acinar), leukocyte infiltration from 0−4 points (0: none; 1: patchy interlobular; 2: moderate diffuse interlobular; 3: mild diffuse interlobular; 4: diffuse interlobular and intra-acinar). Percentage of acinar cell necrosis was also evaluated.

### Laboratory Measurements

Serum amylase activity was measured on a Fluorostar Optima plate reader (BMG Labtech, Ortenberg, Germany) with a colorimetric kinetic method using a commercial kit purchased from Diagnosticum Zrt. (Budapest, Hungary). To evaluate tissue water content, wet weight (WW) of the pancreas was measured right after the *in vivo* experiment, then it was dried for 24 h at 100°C. After that, dry weight (DW) was measured as well. The wet/dry weight ratio was calculated as: [(WW-DW)/WW]×100. Pancreatic myeloperoxidase (MPO) activity, a hallmark of leukocytic infiltration, was measured according to Kuebler et al. (23) and was normalized to total protein content as measured by the Lowry method (24). To determine the extent of inflammatory response in the pancreata, we measured interleukin-1β (IL-1β) levels by a commercial ELISA kit from R&D Systems (Minneapolis, MN, USA) as described by the manufacturer. Blood pH, HCO_3_^-^, and partial pressure of CO_2_ (pCO_2_) in femoral arterial blood samples were measured with a blood gas analyzer (AVL Compact 2, Graz, Austria; (25).

Pancreatic HSP72 expression was measured from tissue homogenate using Western blot analysis (26). Briefly, pancreatic tissue was homogenized with sonication (Branson Sonifer 250; Emerson Electric, Brookfield, CT, USA) on ice in a buffer containing: 10 mM Na-HEPES, 1µM MgCl_2_, 10mM KCl, 1mM DL-dithiothreitol, 5mM iodoacetamide, 4 mM benzamidine-HCl, 1mM phenylmethyl sulfonylfluoride. Protein concentration of the homogenate was determined by the Bradford protein assay. Forty micrograms of protein were loaded per lane. Samples were electrophoresed on an 8% sodium dodecyl sulfate-polyacrylamide gel. The gels were either stained with Coomassie brilliant blue (to demonstrate equal loading of proteins for Western blot analysis) or transferred to a nitrocellulose membrane for 1h at 100V. Membranes were blocked in 5% non-fat dry milk for 1 h and incubated with rabbit anti-HSP72 (1:2500 dilution; a generous gift from István Kurucz, Biorex Laboratories, Veszprém, Hungary, that has been characterized previously; (27) antibody for an additional 1h at room temperature. The immunoreactive protein was visualized by enhanced chemiluminescence, using horseradish peroxidase-coupled anti-rabbit immunoglobulin at 1:5000 dilution (Agilent Technologies, Santa Clara, CA, USA). Quantitative analysis of results was achieved using ImageJ software (NIH, Bethesda, MD, USA). The blot images were cropped, and only the relevant bands are shown in the figures (the raw blot images are presented in the **Supplementary Materials**).

### Measurement of Circulation (Hemodynamics and Pancreatic Microcirculation)

Animals were anaesthetized with sodium pentobarbital (50 mg/kg) i.p. 24 h after the injection of LO and placed in a supine position on a heating pad. Tracheostomy was performed to facilitate spontaneous breathing, and the right jugular vein was cannulated with PE50 tubing for fluid administration such as Ringer’s lactate infusion (10 ml kg^-1^ h^-1^) during the experiments. A thermistor-tip catheter (PTH-01; Experimetria Ltd., Budapest, Hungary) was positioned into the ascending aorta through the right common carotid artery to measure cardiac output (CO) by a thermodilution technique, using a SPEL Advanced Cardiosys 1.4 computer (Experimetria Ltd., Budapest, Hungary). The left common carotid artery was dissected free and an ultrasonic flow-probe (1RS; Transonic Systems Inc., Ithaca, NY, USA) was placed around the exposed artery to measure carotid artery flow. The right femoral artery was cannulated with PE40 tubing to collect arterial blood for pH measurements (25). Carotid artery flow (T206 Animal Research Flowmeter; Transonic Systems Inc.) and pressure (BPR-02 transducer; Experimetria Ltd., Budapest, Hungary) were measured continuously and registered with a computerized data-acquisition system (Experimetria Ltd., Budapest, Hungary).

After median laparotomy, the pancreas was carefully placed on the detector from the abdomen without disturbing the circulation. The pancreas was kept moist with wet gauze. The microcirculation of the pancreas was continuously visualized with intravital orthogonal polarization spectral imaging technique (Cytoscan A/R, Cytometrics, Philadelphia, Pennsylvania, USA). This technique utilizes reflected polarized light at the wavelength of the isobestic point of oxy-and deoxyhaemoglobin (548 nm). As polarization is preserved in reflection, only photons scattered from a depth of 2−300 μm contribute to image formation. A 10x objective was placed onto the serosal surface of the pancreas, and microscopic recordings were made with an S−VHS video recorder 1 (Panasonic AG−TL 700, Matsushita Electric Ind. Co. Ltd, Osaka, Japan). Quantitative assessment of the microcirculatory parameters was performed off-line by frame-to-frame analysis of the videotaped images. Red blood cell velocity (RBCV, mm/s) changes in the postcapillary venules were determined in three separate fields by means of a computer-assisted image analysis system (IVM Pictron, Budapest, Hungary). All microcirculatory evaluations were performed by the same investigator (18).

### Total RNA Isolation and Reverse Transcription Polymerase Chain Reaction

Total RNA was isolated from the control rat brain cortex and pancreas by using TRI Reagent (Molecular Research Center, USA) and 1 μg RNA from each sample was transcribed to complementary DNA by Maxima First Strand cDNA Synthesis Kit (Thermo Fisher, Waltham, MA, USA), according to the manufacturer’s instructions. Gene-specific and exon/exon junction spanning oligonucleotide primer pairs (**Table 1**) were designed with The Universal Probe Library Assay Design Center (Merck KGaA, Darmstadt, Germany). Primers for hypoxanthine phosphoribosyltransferase (HPRT) gene were used as loading control (**Table 1**). PCR was performed with DreamTaq DNA Polymerase (Thermo Fisher) in BioRad C1000 ThermalCycler (Bio-Rad Laboratories, Hercules, CA, USA). After heat inactivation for 3 min at 95°C, cycling conditions were the following: denaturation for 10 s at 95°C, annealing for 10 s at 50°C, polymerization for 10 s at 72°C (40 cycles), final extension for 3 min at 72°C. Products were analyzed on 3% MetaPhor agarose gel (Lonza, Basel, Switzerland), then isolated fragments were sequence verified by capillary DNA sequencing.

**Table 1:** Primers used in this study.

Primer		Sequence	Product size (bp)	Gene ID
**GluN1/** **NMDAR1**	fwd	tgtcatcccaaatgacagga	108	24408
	rvs	ggctcttggtggattgtcac		
**HPRT**	fwd	gaccggttctgtcatgtcg	61	24465
	rvs	acctggttcatcatcactaatcac		

### Immunofluorescent Stainings for N-Methyl-D-Aspartate Receptor and Amylase

Pancreata embedded in cryomatrix were cut into 7 µm thick slices at −20°C with a Leica Cryostat (Leica Biosystems, Buffalo Grove, IL, United States). Slides were kept at −20°C until processing. Immunofluorescent staining was performed in a humidified chamber at room temperature. Sections were fixed in 4% PFA−PBS for 15 min then washed in 1x Tris buffered saline (TBS) for 5 mins, repeated 3 times. Antigen retrieval was performed in Sodium Citrate - Tween 20 buffer (0.001 M Sodium Citrate Buffer, pH 6.0 and 0.05% Tween 20) at 90-96°C for 30 min. After cooling to room temperature in 1x TBS, sections were blocked with 0.01% goat serum and 5x BSA−TBS (bovine serum albumin in Tris Saline Buffer) for 1 h. Thereafter, pancreatic sections were incubated with anti-NMDAR1 rabbit monoclonal antibody (1:100, ThermoFisher Scientific, Waltham, USA) overnight at 4°C in a humidified chamber. The following day slides were washed 3 x 5 min in 1x TBS, then Alexa Fluor 568 goat anti-rabbit secondary antibody was added (1:500) and slides were incubated for 3 h at room temperature, covered from light. After that, co-immunostaining was performed with anti-amylase mouse monoclonal antibody (1:200) and Alexa Fluor 488 goat anti-mouse secondary antibody (1:500) as described above. Samples were washed 3 x 5 min with 1x TBS, then nuclei were counterstained with 2.5 µg/ml Hoechst 33342. After washing 3 times in 1x TBS, Fluoromount Aqueous mounting medium was added. Slides were covered, then left to dry in a dark slide box. After drying, slides were stored at 4°C until visualizing with confocal microscopy (ZEISS LSM 880), and images were processed with ImageJ software (NIH, Bethesda, MD, USA). For proper visibility images were cropped from the raw images and all of them were adjusted uniformly, brightness was increased by 20% with PowerPoint software (Microsoft, Redmond, WA, US). Raw images are shown in **Supplementary Materials**.

### Pancreatic Acinar Cell Isolation

Rat pancreatic acinar cells were isolated with collagenase digestion technique according to Pandol et al. (28). Briefly, animals were sacrificed, and the pancreas was removed, washed, and placed into ice-cold PS, then the tissue was cleaned from fat and lymph nodes. The extracellular solution, used in the next steps contained (in mM) 120 NaCl, 5 KCl, 25 HEPES, 2 NaH_2_PO_4_, 2 CaCl_2_, 1 MgCl_2_, 5 pyruvate, 4 Na-fumarate, 4 Na-glutamate, 12 D-glucose, as well as 0.02% (wt/vol) soybean trypsin inhibitor, 0.2% (wt/vol) bovine serum albumin, 0.025% (vol/vol) minimal essential amino acids and 0.01% (vol/vol) vitamins eagle. After cleaning, the pancreas was cut into small pieces in 5 ml extracellular solution, containing 80 U/ml type 4 collagenase (Worthington Biochemical Co., Lakewood, USA). The tissue was incubated in a shaking water bath at 37°C for 2 x 20 min. After 20 min, the supernatant was removed and 5 ml fresh collagenase solution was added to the tissue fragments. After digestion, acinar cells were washed three times with extracellular solution, then resuspended in Medium 199 solution and placed in 37°C CO_2_ incubator for 15 min. Acini were used for experiments immediately thereafter.

### Acinar Viability

Isolated pancreatic acinar cells were placed into a 96-well plate and 1 µM propidium-iodide (PI) was added to each well. Fluorescence intensity was measured at excitation and emission wavelengths of 540 nm and 620 nm with Fluorostar Optima plate reader every 5 min. The 300 mg/kg dose of KYNA used in the *in vivo* experiments was converted to an equimolar concentration (250 μM). After intensity stabilized (in approximately 1 h), the cells were treated with 20 mM LO, 25-2500 µM KYNA/SZR-72/NMDA according to the experimental protocol. At the end of the experiment (approximately 10 h), Triton X-100 was added to each well to kill every living cells. Intensity measured at this point was considered to represent 100% toxicity. Data were evaluated by selecting minimum (MIN) and maximum (MAX) intensities in each treatment-group. The percentage of cell death at each time point was calculated using the following formula: [(intensity-MIN)/(MAX-MIN)]*100. Figures show the values measured at 8 h.

### Neutrophil Granulocyte Isolation and Measurement of H_2_O_2_ Production

Neutrophil granulocytes were isolated from rats treated with PS, LO or LO+300 mg/kg SZR-72 24 h prior to AP induction using Ficoll-Hypaque density gradient centrifugation. After sacrifice, blood was collected in EDTA coated tubes from each animal. Blood was gently mixed with equal volume of 3% Dextran solution and left to sediment for 40 min. In a conical tube, the leukocyte-rich plasma was carefully added on top of Ficoll-Hypaque, forming two phases. After centrifugation (250 RCF, 40 min) polymorphonuclear and red blood cell pellet was obtained. Erythrocytes were lysed with 0.2 % NaCl solution for no more than 30 sec. Immediately thereafter, lysis was stopped with ice-cold 3% NaCl solution. If red color was still visible after centrifugation, the process was repeated. Granulocytes were resuspended in phosphate buffered saline (PBS) containing 10 mM glucose, then cells were counted in a Bürker chamber. Cell number was adjusted to 1.5x10^4^/100µl. H_2_O_2_ production was measured with Fluorostar Optima plate reader (BMG Labtech, Ortenberg, Germany) using Amplex Red Hydrogen Peroxide/Peroxidase Assay Kit described by the manufacturer.

### Measurement of IL-1β Production in Isolated Acinar Cells

Isolated pancreatic acinar cells were placed into 6-well plates and treated with medium, LO (20mM), KYNA (250µM), SZR-72 (250µM) or with the combination of LO and KYNA/SZR-72 for 6 h. Then cells were washed with PBS, then the washing buffer was removed and cells were frozen at -80°C until further processes. Then 200 µL homogenization buffer (Na-HEPES 10 mM, MgCl_2_ 1µM, KCl 10mM, iodoacetamide 5mM, benzamidine-HCl 4 mM, DL-Dithiothreitol 1mM, Phenylmethyl sulfonylfluoride 1mM) was added to the first well and cells were scratch from the bottom. Further 100 µL homogenization buffer was used to collect the remaining cells. After that the collected suspension of cells was added to the next well (same treated group) and scratching process were repeated. 50 µL cell free homogenization buffer was used to wash and collect the remaining cells. With this process two wells were pooled into one microcentrifuge tube. Following this, 3x15s homogenization with sonication was carried out, and homogenate was incubated for 20 min at 0°C. Then homogenates were centrifuged with 20000 rcf at 4°C for 20 min and supernatants were kept for further measurements. To determine the extent of inflammatory response in the acinar cells interleukin-1β (IL-1β) levels were measured by a commercial ELISA kit from R&D Systems as described by the manufacturer.

### Statistical Analysis

Data are presented as means ± SEM. Experiments were evaluated by one-way ANOVA followed by Holm-Sidak *post hoc* test or two-way ANOVA followed by Bonferroni *post hoc* test (SPSS, IBM, Armonk, NY, USA). P<0.05 was accepted as statistically significant.

## Results

### Dose-Dependent Effects of KYNA and Its Analog SZR-72 on the Severity of AP

**Figure 2:**
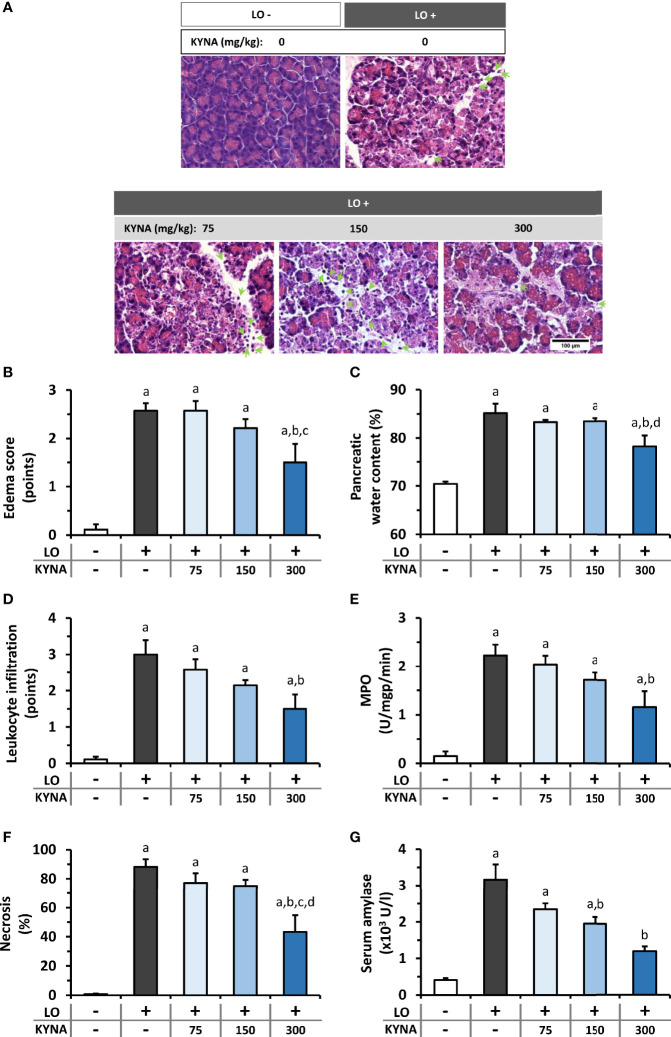
The effects of kynurenic acid (KYNA) on the severity of acute pancreatitis (AP). **(A)** Representative histopathological images of pancreatic tissues of the treatment groups, arrows indicate neutrophil granulocytes. Bar charts show the extent of pancreatic **(B)** edema, **(C)** water content, **(D)** leukocyte infiltration, **(E)** myeloperoxidase (MPO) activity, **(F)** necrosis, and **(G)** serum amylase activity measurements. Values represent means with standard error, n=5-14. One-way ANOVA was performed followed by Holm-Sidak post-hoc test. Statistically significant differences (p<0.05) were marked with: (a) *vs*. control; (b) *vs*. LO; (c) *vs*. LO+75 mg/kg KYNA; (d) *vs*. LO+150 mg/kg KYNA. AP, acute pancreatitis; KYNA, kynurenic acid; LO, L-ornithine-HCl; MPO, myeloperoxidase.

Three different doses of KYNA were tested to determine its effects on LO-induced AP (**Figure 2**). Representative histological images show morphological changes of the pancreas in different groups (**Figure 2A**). LO administration alone induced necrotizing AP, while 300 mg/kg KYNA significantly reduced pancreatic tissue injury observed in AP. Marked increase of pancreatic edema was detected in the LO-treated groups compared to control, while the highest dose of KYNA (300 mg/kg) significantly reduced it (**Figures 2B, C**). Leukocyte infiltration into the pancreas and tissue MPO activity also significantly increased in AP groups compared to control (**Figures 2D, E**). As seen in case of edema, the 300 mg/kg dose of KYNA significantly reduced both leukocyte infiltration and MPO activity during AP, while smaller doses of KYNA were ineffective. The most important measure of inflammation is tissue damage, which was remarkable in response to a single LO-treatment (**Figure 2F**), but it significantly decreased in the 300 mg/kg KYNA group. Serum amylase activity also increased in the LO group, while 150 and 300 mg/kg KYNA significantly reduced the enzyme activity (**Figure 2G**). Overall, the two lower doses (75 and 150 mg/kg) of KYNA did not significantly influence most of the measured values, but 300 mg/kg KYNA reduced the severity of AP.

**Figure 3:**
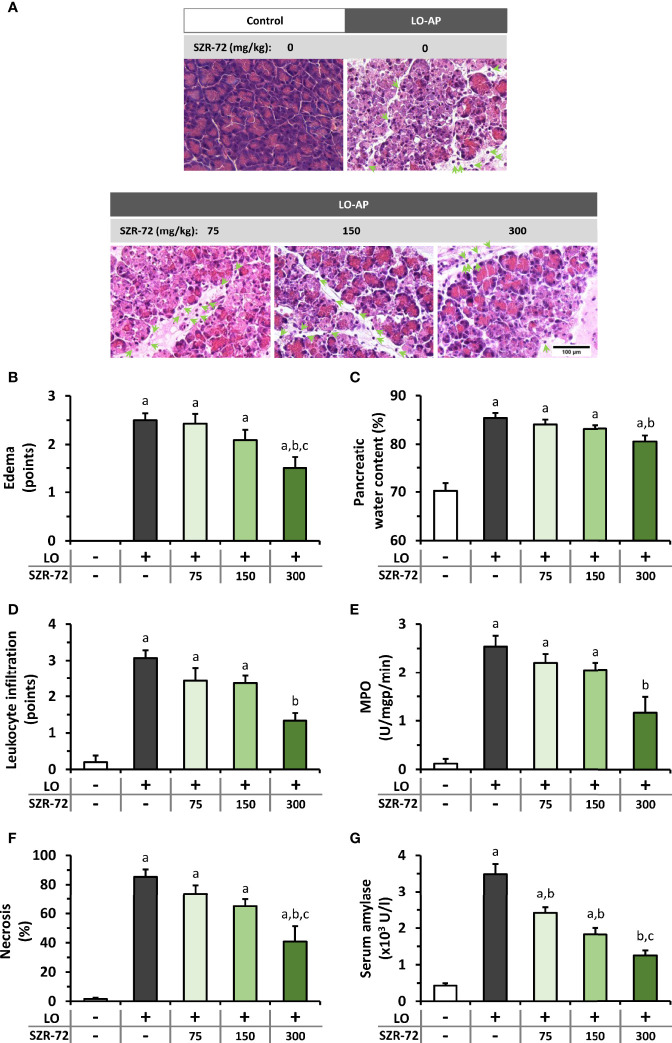
The effects of SZR-72 on the severity of AP. **(A)** Representative histopathological images of pancreatic tissues of the treatment groups, arrows indicate neutrophil granulocytes. Bar charts show the extent of pancreatic **(B)** edema, **(C)** water content, **(D)** leukocyte infiltration, **(E)** myeloperoxidase (MPO) activity, **(F)** necrosis, and **(G)** serum amylase activity measurements. Values represent means with standard error, n=5-11. One-way ANOVA was performed followed by Holm-Sidak post-hoc test. Statistically significant differences (p<0.05) were marked with: (a) *vs*. control; (b) *vs*. LO; (c) *vs*. LO+75 mg/kg SZR-72. LO, L-ornithine-HCl; MPO, myeloperoxidase.

Similar results to KYNA were obtained when the effects of different doses of its analog, SZR-72 were examined (**Figure 3**). Histological images show the effects of LO and the co-treatment of LO and SZR-72 (**Figure 3A**). The signs of AP could be observed in tissue sections and 300 mg/kg SZR-72 reduced tissue damage. The 300 mg/kg dose of SZR-72 was able to significantly reduce the AP-evoked increases in pancreatic edema and leukocyte infiltration (**Figures 3B, D**). These results were supported by measurements of pancreatic water content and MPO activity (**Figures 3C, E**). The scores of pancreatic damage could be significantly reduced by the highest dose of SZR-72 in AP (**Figure 3F**). Serum amylase activity increased in response to LO injection, which was decreased by all SZR-72 doses (**Figure 3G**). Overall, AP severity parameters were reduced by 300 mg/kg SZR-72 treatment.

### The Effects of KYNA and SZR-72 Treatment on Microcirculation and Hemodynamic Changes in AP

**Figure 4:**
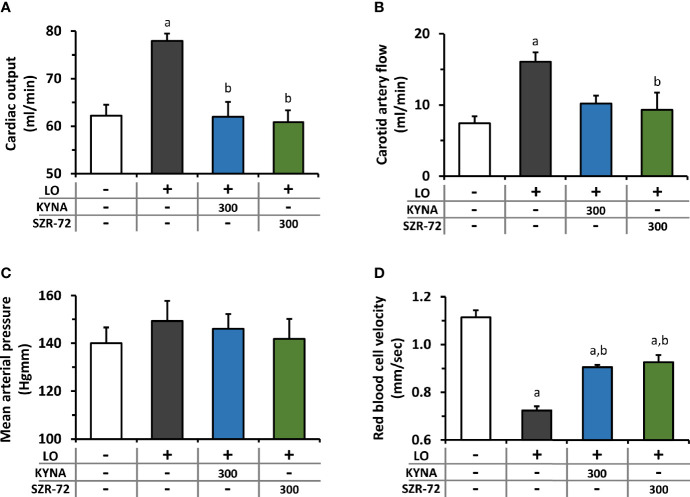
Changes in circulation and haemodynamic parameters during experimental AP and treatments with KYNA and SZR-72. Bar charts show **(A)** cardiac output, **(B)** carotid artery flow **(C)** mean arterial pressure, and **(D)** red blood cell velocity. Values represent means with standard error, **(A–C)** n=3-6; **(D)** n=60-98. One-way ANOVA was performed followed by Holm-Sidak post-hoc test. Statistically significant differences (p<0.05) were marked with: (a) *vs*. control; (b) *vs*. LO. LO, L-ornithine-HCl.

Hemodynamic parameters were determined during AP and KYNA/SZR-72 treatments (**Figure 4**). AP significantly increased cardiac output and carotid artery flow compared to the control animals (**Figures 4A, B**). Cardiac output in rats with AP was reduced to the level of the control group by both KYNA and SZR-72 compounds, whereas the decrease in carotid artery flow was significant only in case of SZR−72. Mean arterial blood pressure was comparable in each experimental group (**Figure 4C**). Pancreatic microcirculation was quantified by measuring serosal RBCV (**Figure 4D**). Interestingly, microcirculation significant decreased in LO−induced AP compared to the control group. However, pre-treatment with KYNA or SZR-72 (300 mg/kg) was able to improve microcirculation during AP.

**Figure 5:**
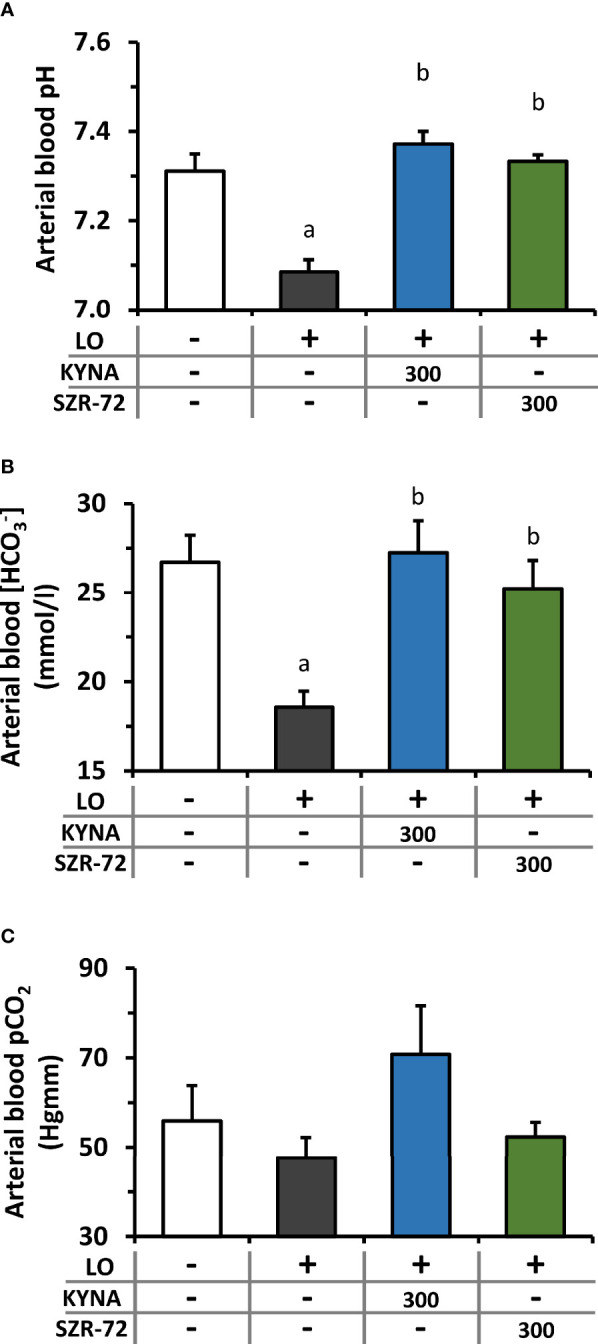
Plasma pH and HCO_3_^-^ and pCO_2_ levels during experimental AP and treatments with KYNA and SZR-72. Bar charts show **(A)** arterial blood pH, **(B)** arterial blood HCO_3_^-^ concentration, and **(C)** arterial blood CO_2_ pressure. Values represent means with standard error, n=3-6. One-way ANOVA was performed followed by Holm-Sidak post-hoc test. Statistically significant differences (p<0.05) were marked with: (a) *vs*. control; (b) *vs*. LO. LO, L-ornithine-HCl.

LO−induced AP caused a significant drop in arterial blood pH and bicarbonate concentration resulting in metabolic acidosis, which was restored to the level of the control group by KYNA and SZR-72 pre-treatments (**Figures 5A, B**). At the same time, there was no detectable difference in arterial pCO_2_ between the examined groups (**Figure 5C**).

### Changes in Pancreatic IL-1β and HSP72 Expression in AP Upon KYNA and SZR-72 Treatment

**Figure 6:**
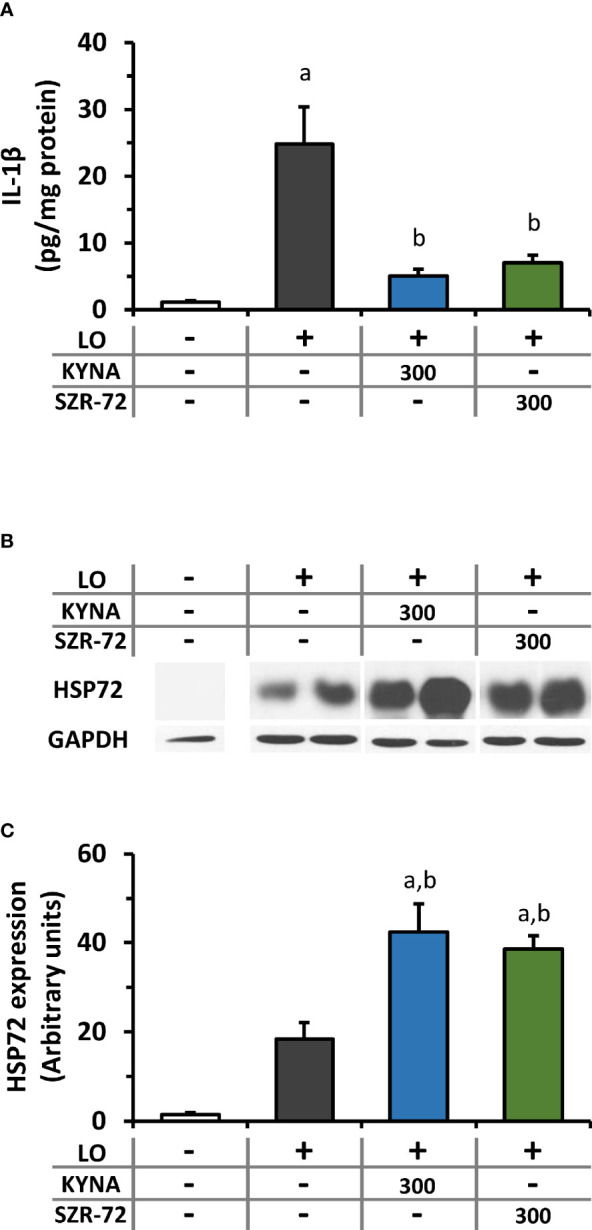
Changes in interleukin 1 beta (IL-1β) and heat shock protein 72 (HSP72) levels in AP in rats treated with 300 mg/kg KYNA or SZR-72. **(A)** Pancreatic IL-1β level, **(B)** representative Western blot images of pancreatic HSP72 and glicerinaldehide-3-phosphate-dehydrogenase (GAPDH) levels, and **(C)** densitometry of Western Blot images for pancreatic HSP72 level. Values represent means with standard error, n=7-10. One-way ANOVA was performed followed by Holm-Sidak post-hoc test. Statistically significant differences (p<0.05) were marked with: (a) *vs*. control; (b) *vs*. LO.

KYNA and SZR-72 alone did not affect pancreatic IL-1β content of the pancreas (**Supplementary Figure 1A**). However, IL-1β levels significantly increased in the LO groups compared to control animals (**Figure 6A**). In the AP groups that received KYNA or SZR-72, IL-1β levels were significantly reduced and reached the level of control.

As a member of the HSP70 family, HSP72 is the major stress-induced protective chaperone in mammalian cells. First, we examined how KYNA and SZR-72 treatment affected pancreatic HSP72 levels in physiological conditions (**Supplementary Figures 1B, C**) and during AP (**Figures 6B, C**; the corresponding raw blot image is shown in **Supplementary Figure 2**). KYNA and SZR-72 alone significantly increased HSP72 expression compared to the control group, and SZR-72 had more prominent effect on HSP72 protein expression than KYNA (**Supplementary Figures 1B, C**). In our experiments, it was clear that the level of HSP72 was elevated in AP compared to the control group (**Figures 6B, C**). However, when the animals also received KYNA or SZR-72 pre-treatment, the amount of HSP72 significantly increased even compared to the AP group without KYNA or SZR-72.

### The Detection of NMDA Receptor-1 in the Pancreas

**Figure 7:**
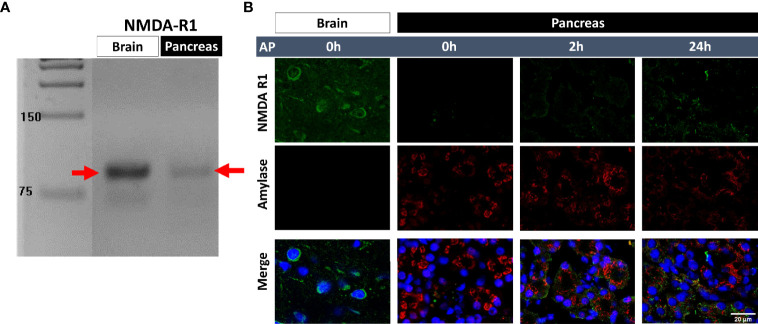
Detection of N-methyl-D-aspartate receptor 1 (NMDAR1) expression. **(A)** NMDAR1 mRNA expression in brain cortex and pancreas, **(B)** representative immunofluorescent images (NMDAR1, amylase, and cellular nuclei stainings) of pancreatic tissue (scale bar: 20 µm). Panc, pancreas.

NMDAR1 expression was examined by RT-PCR and immunohistochemistry (**Figure 7**). In both methods, brain tissue was used as a positive control. mRNA expression was much lower in the pancreas than in the brain (**Figure 7A**; full scan of the original gel is shown in **Supplementary Figure 3**). This was also confirmed by immunohistochemistry, where NMDAR1 staining of the brain was clearly visible (**Figure 7B**; raw images are presented in **Supplementary Figure 4**). The image of the control pancreas showed low NMDAR1 expression with well-structured amylase staining. The pancreas was sampled 2 and 24 h after LO administration in order to visualize if there was a difference in NMDAR1 staining depending on how advanced the inflammation was. NMDAR1 staining was found to be more pronounced 2 h after AP induction, however, the strongest staining was observed after 24 h. In parallel, amylase staining lost its structural integrity as the inflammation progressed.

### *In Vitro* Protective Effects of KYNA, SZR-72, and NMDA on LO-Induced Cellular Toxicity

**Figure 8:**
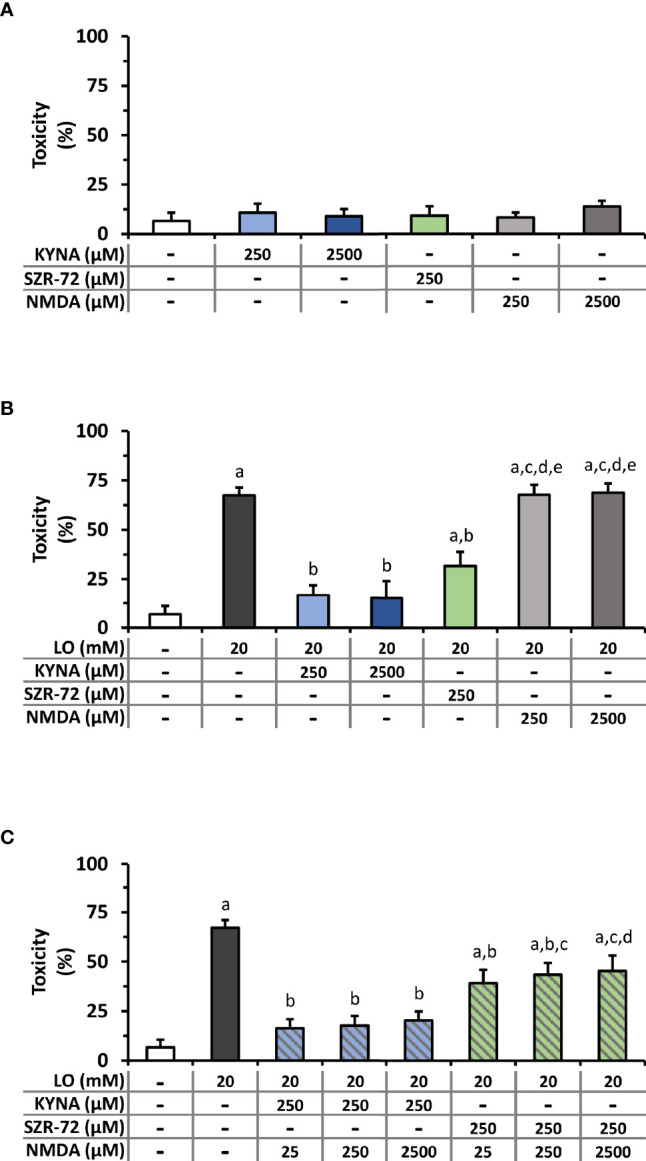
Toxicity measurements on isolated pancreatic acinar cells. **(A)** Toxicity of KYNA, SZR-72, and N-methyl-D-aspartate (NMDA) in different concentrations. **(B)** Toxic effect of L-ornithine-HCl (LO) combined with KYNA, SZR−72 or NMDA treatments. One-way ANOVA was performed followed by Holm-Sidak post-hoc test. Statistically significant differences (p<0.05) were marked with: (a) *vs*. control; (b) *vs*. LO; (c) *vs*. LO+250 µM KYNA; (d) *vs*. LO+2500 µM KYNA; (e) *vs*. LO+250 µM SZR-72. **(C)** Toxicity of co-treatment of LO (20 µM) and NMDA (25, 250, 2500 µM) combined with 250 µM KYNA or SZR-72. One-way ANOVA was performed followed by Holm-Sidak post-hoc test. Statistically significant differences (p<0.05) were marked with: (a) *vs*. control; (b) *vs*. LO; (c) *vs*. LO+250 µM KYNA+25 µM NMDA; (d) *vs*. LO+250 µM KYNA+250 µM NMDA. Values represent means with standard error, n=4-10.

The effects of KYNA and SZR-72 were measured on LO-induced cellular toxicity in *in vitro* experiments. Since both compounds are NMDAR antagonists, NMDA was also applied to reveal whether KYNA or SZR-72 exert their effect on NMDAR. Before testing the protective properties of KYNA and SZR-72, or their interaction with NMDAR, the safe concentrations of KYNA, SZR-72, and NMDA were determined on isolated pancreatic acinar cells (**Figure 8A** and **Supplementary Figure 5**). SZR-72 could be safely administered until 625 µM, higher concentrations were toxic to acinar cells (**Supplementary Figure 5**). As the 300 mg/kg dose of KYNA proved to be effective *in vivo*, the corresponding, equimolar (250µM) and ten times higher concentrations (2500 μM) of KYNA and NMDA were tested on acini. In case of SZR-72, only the 250 µM concentration was used in further viability studies because the ten times higher concentration has been already proved to be toxic. Neither KYNA nor NMDA affected pancreatic acinar viability even at a concentration of 2500 µM (**Figure 8A**). We then measured the effect of LO treatment on cell viability and whether it could be affected by KYNA, SZR-72, or NMDA (**Figure 8B**). LO was shown to be highly toxic to pancreatic acinar cells. However, KYNA prevented the toxic effect of LO at both 250 and 2500 μM concentrations and cell viability was comparable to the control group. Treatment with 250 μM SZR-72 also significantly reduced LO-induced toxicity. NMDA did not affect the toxicity of LO at any concentrations. Last, we examined whether the beneficial effects of KYNA and SZR-72 could be suspended by the addition of NMDA (**Figure 8C**). Beside LO, acinar cells received 250 μM KYNA or SZR-72 and increasing doses of NMDA (25, 250, 2500 μM). Co-treatment with NMDA had no effect on cell viability. KYNA and SZR-72 were still able to significantly reduce toxicity compared to the LO group. Moreover, KYNA treatment resulted in decreased cellular toxicity which was comparable to the control group.

### SZR-72 Reduces the Activity of H_2_O_2_ Production in Isolated Neutrophil Granulocytes, But Has No Effect on IL-1β Expression of Pancreatic Acinar Cells

**Figure 9:**
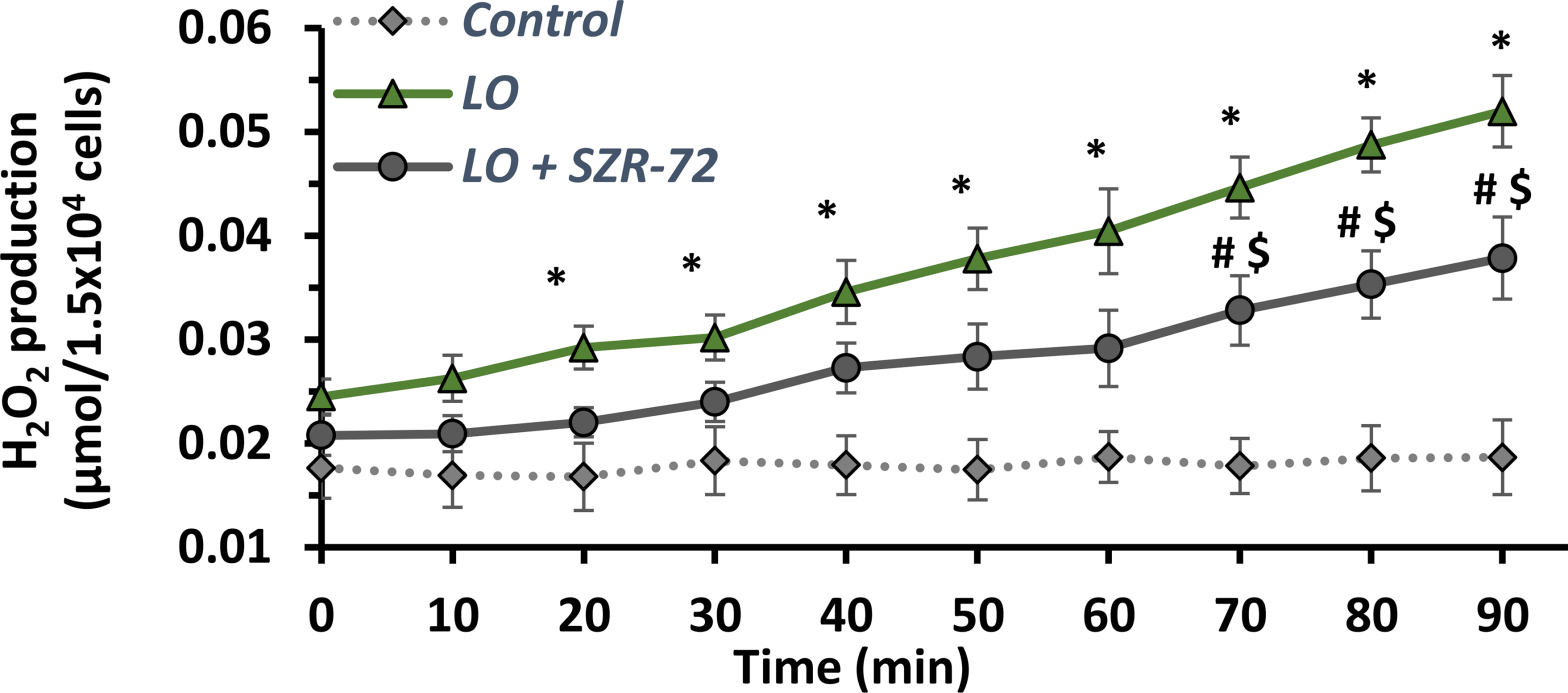
Time course of H_2_O_2_ production of neutrophil granulocytes isolated from rats treated with physiological saline, LO or LO+300 mg/kg SZR-72. Values represent means with standard error, n=4. Two-way ANOVA was performed followed by Bonferroni post-hoc test. Statistically significant differences (p<0.05) were marked with: (*) control *vs*. LO; (#) LO *vs*. LO+SZR-72; ($) control *vs*. LO+SZR-72.

Neutrophil granulocytes play an important role in the development of AP. H_2_O_2_ production corresponds to their function. The effect of SZR-72 was determined on neutrophil granulocyte function (**Figure 9**). H_2_O_2_ production of granulocytes was examined after cell isolation from control, LO- and LO + SZR-72-treated animals. In case of control granulocytes, H_2_O_2_ production remained at baseline throughout the experiment. In contrast, neutrophils from AP animals produced increased amounts of H_2_O_2_, the level of which was significantly different from the control group from as early as 20 min. However, when neutrophils from LO and SZR-72 co-treated animals were examined, a significant decrease was observed in H_2_O_2_ production from 70 min compared to LO treatment alone.

The IL-1β protein expression of isolated acinar cells was measured *in vitro* after 6h treatment with LO, KYNA, and/or SZR-72 (**Supplementary Figure 6**). LO administration did not induce any change in IL-1β expression compared to the control group in acinar cells. Furthermore, KYNA, SZR-72 or their combinations with LO did not affect the proinflammatory cytokine production, these were comparable with the control group.

## Discussion

As AP is a disorder without specific therapy, it is important to find possibilities for its management. The pathophysiology of the disease involves multiple cell types and processes (6). The pathway of tryptophan metabolism is unambiguously disturbed during AP, resulting in overactivation of kynurenine-3-monooxygenase enzyme and excess production of pro-inflammatory 3-HK (20, 21). In this study, we tested the possible application of endogenous tryptophan pathway metabolite KYNA, and its synthetic derivative SZR-72 for the treatment of experimental AP. Our novel findings with KYNA or SZR-72 administration in experimental AP are the following: They (1) dose-dependently reduced the severity of the disease; (2) reduced the proinflammatory cytokine IL-1β expression *in vivo*; (3) increased the synthesis of HSP72; (4) reduced the extent of metabolic acidosis; (5) restored pancreatic microcirculation; (6) suppressed the function of neutrophil granulocytes. (7) In addition, their effect was likely to be independent of acinar NMDAR1.

SZR-72 can cross the blood-brain barrier, while KYNA is poorly permeable (14, 15). Therefore, SZR-72 can exert its effect in the central nervous system as well (29, 30). As the results with SZR-72 and KYNA were similar, we do not think that the possible central nervous system effects of SZR-72 play part in the protection of AP.

We demonstrated that the 300 mg/kg dose of KYNA and SZR-72 exerted strong anti-inflammatory effects. Csáti et al. (31) also applied the same dose of KYNA or kynurenic acid amide 2 in rats i.p., and they observed successful suppression of inflammation evoked by trigeminal ganglion activation. Similar results were obtained when SZR-72 was applied at 300 mg/kg dose i.p. in rats, the KYNA analogue exerted anti-inflammatory response in a model of trigeminal nerve activation (32). In case of rat experimental colitis, more than ten times smaller doses could be used effectively (18, 25). Furthermore, Juhász et al. (16) successfully applied KYNA or SZR-72 in a sepsis model at 2 x 15 or 2 x 23.5 mg/kg respectively. Based on these results, it seems that the effective dose of KYNA and SZR-72 also depends on the disease model.

Hemodynamic parameters like cardiac output and arterial blood flow were increased by AP. Interestingly, this was reduced by KYNA/SZR-72 administration. Blood pressure was unchanged; thus, it is likely that the increase in heart rate contribute to the increased cardiac output. The exact mechanism how KYNA and SZR-72 may affect heart rate is unknown, but most probably this effect is indirect. Similar findings were seen by Badzynska et al. (33) in spontaneously hypertensive rats, where treatment with 25 mg/kg/day KYNA decreased heart rate. An explanation can be the effect of pain, as pain is one of the symptoms of AP and it positively relates to heart rate (34). GPR35 receptor was considered important for nociceptive transmission, and through this receptor KYNA can reduce the pain, which can contribute to the reduced heart rate. However, these speculations should be tested in the future.

AP causes the impairment of both pancreatic and systemic microcirculation (35), which are among the early signs of AP (36). We showed significantly decreased RBCV in the pancreas during experimental AP, which was remarkably restored by the administration of KYNA or SZR-72. The reduced organ microcirculation contributes to ischemia and organ failure, not just in the pancreas but in other organs like the kidneys or lungs. Therefore, KYNA or SZR-72 can alleviate the symptoms of multiple and/or persistent organ failure which is present in the severe form of the disease. Furthermore, Zhang et al. (37) found that decrease in intestinal microcirculation secondary to severe AP can lead to reduced mucosal barrier integrity and immunity, thus increased possibility of infection, sepsis, and mortality. Based on this, the beneficial effect of KYNA and SZR-72 on microcirculation is important and should be further investigated. Interestingly, in our earlier work, KYNA improved ileal microcirculation in a sepsis model, while SZR-72 was ineffective (16). However, in that model SZR-72 could improve mitochondrial respiration, resulting in improved conversion of ADP to ATP. In the present research, the investigation of mitochondrial function was not in focus. However, mitochondrial dysfunction is common in AP and has serious effects (6), therefore further studies are also needed to reveal how KYNA or its derivatives modulate that.

AP is often accompanied by acid-base disturbance. Our earlier work showed the relationship between AP severity and metabolic acidosis (38). Meta-analyses of clinical studies confirmed that the severity of AP relates to the extent of metabolic acidosis. Furthermore, experimental AP aggravated the pre-existing acid-base imbalance. There are several mechanisms that trigger metabolic acidosis during AP, e.g. loss of bicarbonate-rich pancreatic juice through pancreatic fistula or drainage, lactic acidosis due to shock or sepsis which can develop in AP (39). An important observation was that both KYNA and SZR-72 effectively restored the decreased pH and HCO_3_^-^ concentration in the plasma. The exact mechanism how they affect the acid-base balance is unknown, but this effect could also contribute to the reduced disease severity.

HSP72 is an inducible chaperon which is upregulated in different conditions of stress like inflammation. It was found earlier that thermal stress-induced HSP72 increase could protect against AP (40, 41), and pharmacological induction of HSP72 by BRX-220 was also effective in treatment of experimental AP (42, 43). Furthermore, overexpression of HSP72 in transgenic mice enhanced recovery from AP (44). In our study, we showed that both KYNA and SZR-72 significantly increased pancreatic HSP72 expression in rats. Pancreatic HSP72 expression was also increased in AP, KYNA or SZR-72 treatment further upregulated protein expression. SZR-72 was significantly more potent HSP72-inducer than KYNA. The effects of KYNA or SZR-72 on HSP72 can be one of the mechanisms how they exert protection in AP.

NMDAR1 receptor expression was present in pancreatic tissue even in physiological conditions. Surprisingly, NMDAR1 protein expression was increased by the progression of AP. This phenomenon could be explained by three reasons (1): pancreatic cells (e.g. acinar, ductal, beta cells) increased their expression of NMDAR1 (2); invading leukocytes express the receptor (3); the previous two together. We tested whether KYNA or SZR-72 exert their effects *via* NMDAR1. *In vitro* acinar cell LO toxicity measurements demonstrated that the observed protection of KYNA or SZR-72 was unlikely to be related to NMDAR1. Receptor agonist NMDA did not influence the effects of receptor antagonists KYNA or SZR-72 even at ten times the concentration. Therefore, the observed protection against LO-AP could be a direct effect or could be mediated by another receptor like GPR35. KYNA is an endogenous antioxidant, and it can decrease ROS release evoked by AP (45). GPR35 receptor is present in macrophages, eosinophil and basophil granulocytes, mast cells, natural killer T cells, and several cells along the digestive tract (46). GPR35 activation will result in decreased intracellular Ca^2+^ and cAMP signals, inhibition of phosphoinositide 3-kinase/protein kinase B and mitogen-activated protein kinase (MAPK) pathways. All these effects of KYNA-GPR35 interactions contribute to immunosuppression. Our goal was not to investigate the GPR35-mediated effect of KYNA or SZR-72 in AP, but further studies can focus on it.

KYNA or SZR-72 markedly reduced the pancreatic IL-1β expression *in vivo*. However, this effect seems to be independent of acinar cells. Therefore, the tested agents most probably affect leukocytes, and this can result in decreased cytokine release from the pancreatic tissue. Neutrophil granulocytes are the first inflammatory cells reaching the pancreas during AP. ROS such as H_2_O_2_ is produced in large quantities by neutrophils which reflects the activity of these cells (47). Our measurements showed that *in vivo* administration of SZR-72 reduced H_2_O_2_ production in neutrophil granulocytes isolated from AP rats. Since neutrophils contribute to AP by amplifying the inflammatory cascade, the reduced activity of these cells by KYNA or SZR-72 is also beneficial and can contribute to their mechanism of action.

In conclusion, we showed that treatment with endogenous tryptophan metabolite KYNA and its synthetic analog SZR-72 dose dependently reduced the severity of experimental AP (**Table 2**). There may be several mechanisms mediating this protective effect. Both molecules reduce the pancreatic expression of the proinflammatory cytokine IL-1β and increase the expression of HSP72 protein. These compounds also ameliorate metabolic acidosis, and restore hemodynamic parameters including pancreatic microcirculation. Their action seems to be independent of acinar NMDAR1 in AP. SZR-72 also suppresses the activation of neutrophil granulocytes. Overall, these molecules could be beneficial in AP.

**Table 2:** Summarizing the effects (*in vivo*: 300 mg/kg; *in vitro*: 250 µM) of KYNA and SZR-72 in AP.

			KYNA	SZR-72
**Experimental AP** **(*in vivo*)**	**Pancreatic effects**	**Histological parameters**	↓↓	↓↓
		**MPO**	↓↓	↓↓
		**Water content**	↓↓	↓↓
		**Local microcirculation**	Partially restored	Partially restored
		**IL-1β expression**	Restored	Restored
		**HSP72 expression** **during AP**	↑↑	↑↑
		**HSP72 expression in physiological conditions**	↑	↑↑
	**Systemic effects**	**Serum amylase**	↓↓	↓↓
		**Cardiac output**	Restored	Restored
		**Metabolic acidosis**	Restored	Restored
***In vitro* experiments**	**Pancreatic acinar cells**	**Cell protection**	++	+
		**IL-1β expression**	no effect	no effect
	**Neutrophil granulocytes**	**Suppression of ROS production**	N.A.	++

Explanation of symbols and phrases: **↓**, decrease; **↑**, increase; +, positive effect; restored/partially restored, the measured condition during AP was restored/partially restored to control levels after KYNA or SZR-72 treatment. AP, acute pancreatitis; HSP72, heat shock protein 72; IL-1β, interleukin-1β; KYNA, kynurenic acid; N.A., not available; MPO, myeloperoxidase; ROS, reactive oxygen species; SZR-72, 2-(2-N,N-dimethylaminoethyl-amine-1carbonyl)-1H-quinolin-4-one hydrochloride.

## Data Availability Statement

The raw data supporting the conclusions of this article will be made available by the authors, without undue reservation.

## Ethics Statement

The animal study was reviewed and approved by Hungarian National Food Chain Safety Office 1024 Budapest, Keleti Károly u. 24. (1525 Budapest, Pf. 30).

## Author Contributions

ZR had the original idea, initiated the study, obtained funding, and supervised the experimental procedures. Most protocols were designed by ZB, EK, LK, and ZR. Animal experiments were performed by ZB, EK, BK, EB, GF, LK (*in vivo* AP experiments), and GV (microcirculation, haemodynamic measurements). *In vitro* measurements were fulfilled by ZB, EK, EO, GF (pancreatic acinar cell isolation, acinar viability, acinar IL-1β measurement), AH (total RNA isolation, RT-PCR) and CP (neutrophil granulocyte isolation, H_2_O_2_ production measurement). BI, LV, FF, AG, VT, MD, TM, VV, JM, and PH provided conceptual advice on experimental design and protocol. ZB and EK performed data analysis, ZB, EK, GF, and LK worked on statistical analysis, ZB, EK, EB, GF, and LK produced the figures. ZB, EK, LK, and ZR wrote the manuscript. All authors reviewed the manuscript and approved the final version.

## Funding

This work was supported by EFOP-3.6.2–16–2017–00006, GINOP-2.3.2-15-2016-00034, János Bólyai Research Grant (BO/00866/20/5), ÚNKP Grant (ÚNKP-20-5-SZTE-163), NKFIH PD129114 and NKFIH K119938, University of Szeged Open Access Fund (Grant No. 5304). The funders did not influence the interpretation of results in any way.

## Conflict of Interest

Author VT is employed by the company Creative Laboratory Ltd., Szeged, Hungary.

The remaining authors declare that the research was conducted in the absence of any commercial or financial relationships that could be construed as a potential conflict of interest.

## Publisher’s Note

All claims expressed in this article are solely those of the authors and do not necessarily represent those of their affiliated organizations, or those of the publisher, the editors and the reviewers. Any product that may be evaluated in this article, or claim that may be made by its manufacturer, is not guaranteed or endorsed by the publisher.
