# Supplementary material for: Kynurenic Acid and Its Analogue SZR-72 Ameliorate the Severity of Experimental Acute Necrotizing Pancreatitis
Source: Front Immunol. 2021 Oct 21;12:702764. doi: 10.3389/fimmu.2021.702764 (PMC8567016; doi:10.3389/fimmu.2021.702764)
Supplement: Supplementary file 1 [file DataSheet_1.pdf]

Supplementary Material

Kynurenic acid and its analogue SZR-72 ameliorate the severity of experimental acute necrotizing pancreatitis

Zsolt Balla[1,2†], Eszter Sára Kormányos[1†], Balázs Kui[3], Emese Réka Bálint[1], Gabriella Fűr[1], Erik Márk Orján[1], Béla Iványi[4], László Vécsei[5,6], Ferenc Fülöp[7,8], Gabriella Varga[9], András Harazin[10], Vilmos Tubak[11], Mária A. Deli[10], Csaba Papp[12,13], Attila Gácser[12,13], Tamara Madácsy[3], Viktória Venglovecz[14], József Maléth[3], Péter Hegyi[3,15,16], Lóránd Kiss[1*], Zoltán Rakonczay Jr.[1]*

1.1 Supplementary Figures

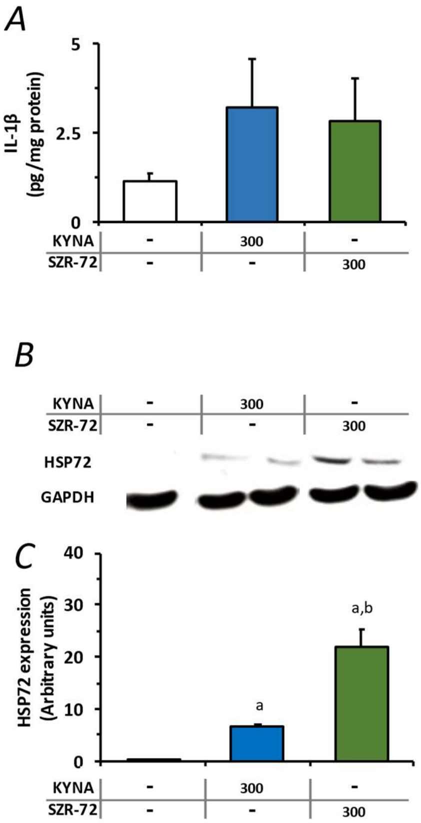

Supplementary Figure 1. Changes in interleukin 1 beta (IL-1β) and heat shock protein 72 (HSP72) levels during treatments with 300 mg/kg KYNA or SZR-72 in rats. **(A)** Pancreatic IL-1β level, **(B)** representative Western blot images of pancreatic HSP72 and glicerinaldehide-3-phosphate-dehydrogenase (GAPDH) levels, and **(C)** densitometry of Western Blot images for pancreatic HSP72 level. Values represent means with standard error, n=7-10. One-way ANOVA was performed followed by Holm-Sidak post-hoc test. Statistically significant differences ($p<0.05$) were marked with: (a) vs. control; (b) vs. KYNA.

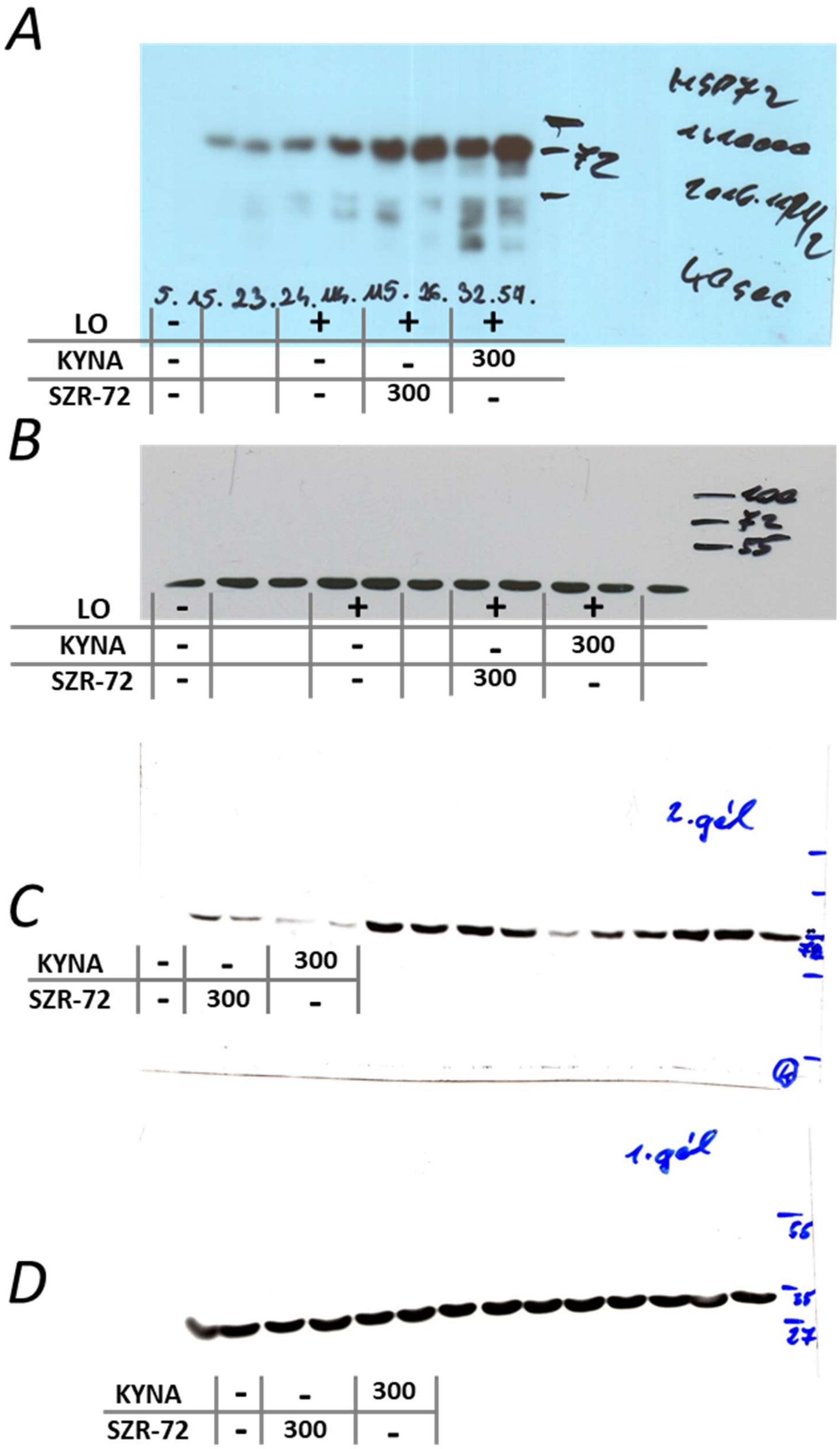

Supplementary Figure 2. Raw images of HSP72 and GAPDH protein expressions. **(A)** HSP72 and **(B)** GAPDH protein expression in AP upon 300 mg/kg KYNA or SZR-72 treatment in rats. Changes in **(C)** HSP72 and **(D)** GAPDH levels during treatments with 300 mg/kg KYNA or SZR-72 in rats without AP.

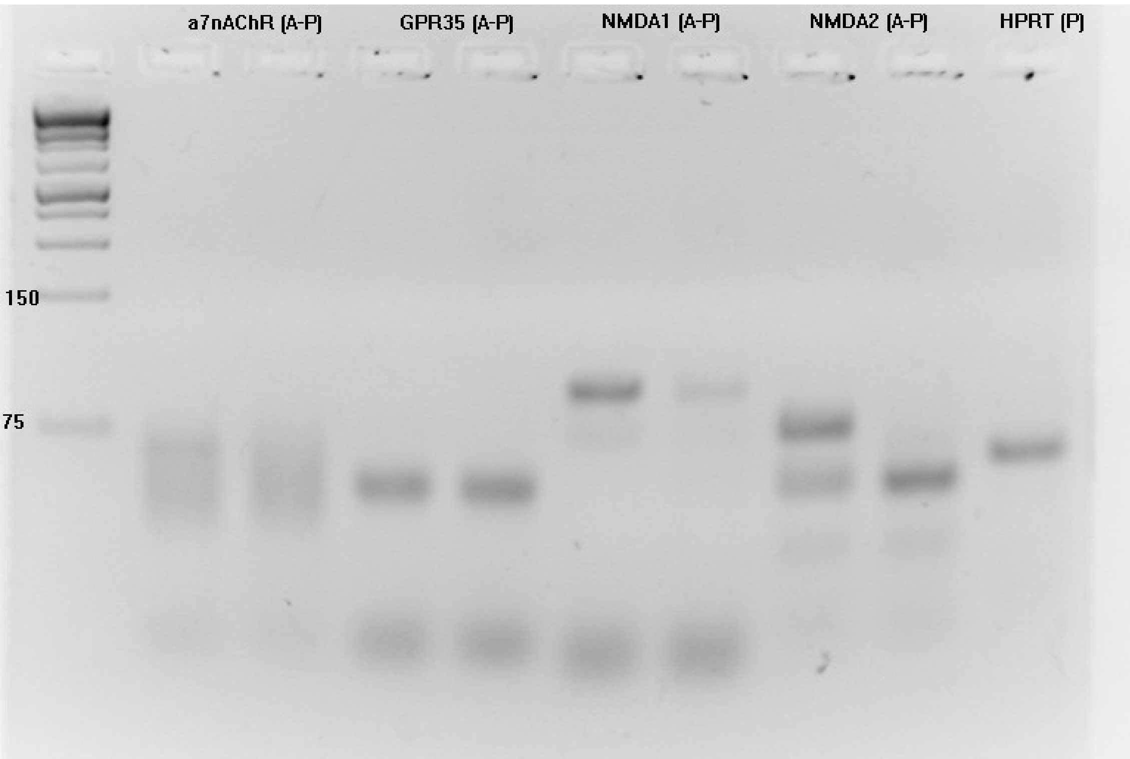

Supplementary Figure 3. Raw blot image of reverse transcription polymerase chain reaction (RT-PCR). The corresponding image is presented in Figure 7.

Abbreviations: *α7nAChR:* alpha 7 nicotinic acetylcholine receptor; GRP35: G protein-coupled receptor; NMDA1: N-methyl-D-aspartate receptor 1; NMDA2: N-methyl-D-aspartate receptor 2; HPRT: hypoxanthine phosphoribosyltransferase; A: brain; P: pancreas.

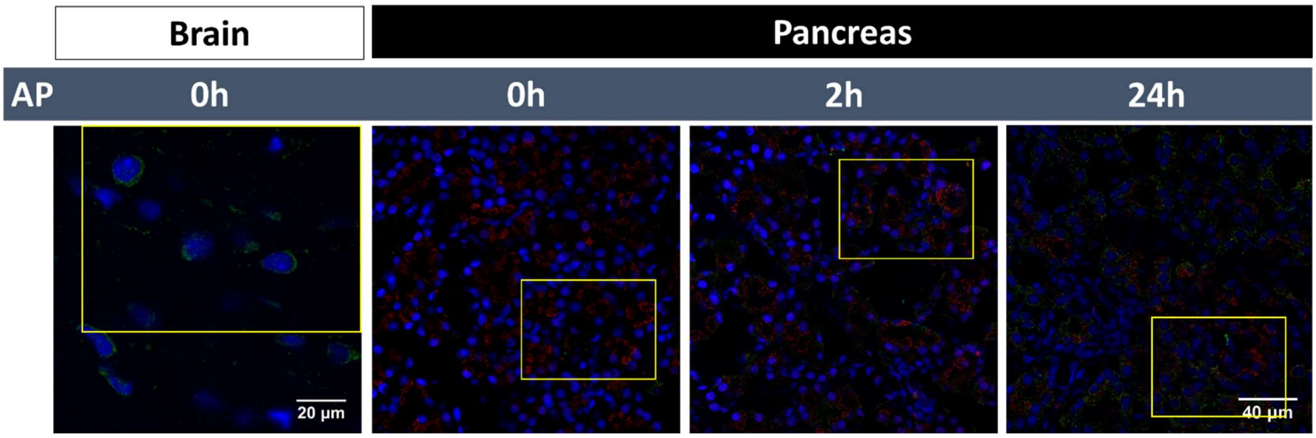

Supplementary Figure 4. Raw Immunofluorescent images of pancreatic tissue and brain cortex. Blue: nuclei; red: amylase; green: N-methyl-D-aspartate receptor 1 (NMDAR1). Yellow squares show the cropped images which is presented in Figure 7.

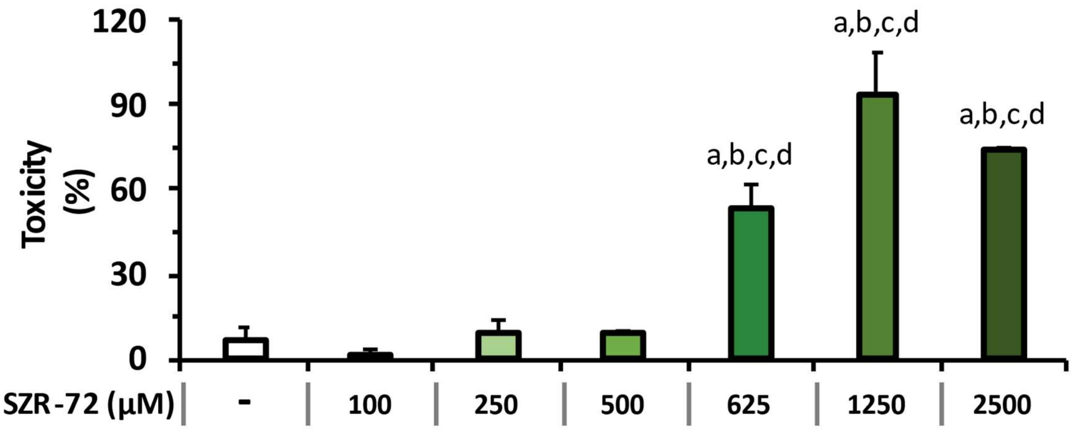

Supplementary Figure 5. Concentration-dependent toxicity of SZR-72. Values represent means with standard error, n=4-10. One-way ANOVA was performed followed by Holm-Sidak post-hoc test, $p<0.05$. (a) vs. control; (b) vs. 100 µM SZR-72; (d) vs. 250 µM SZR-72; (e) vs. 300 µM SZR-72.

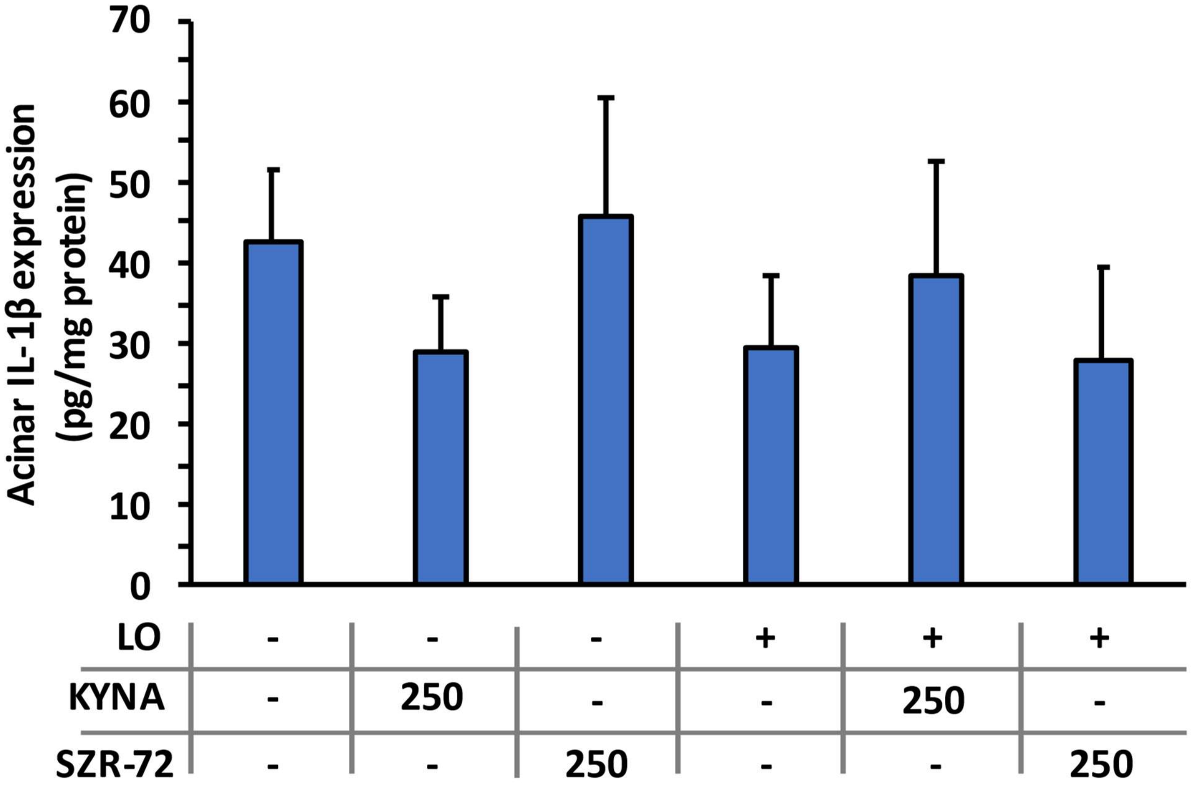

Supplementary Figure 6. Acinar IL-1β concentrations remain unchanged after treatment with L-Ornithine (LO), KYNA, and/or SZR-72. Bar chart shows IL-1β concentrations 6h after the treatments with 20 mM LO, 250 µM KYNA, and/or 250 µM SZR-72 using isolated rat pancreatic acinar cells. Values represent means with standard error, n=7-14. One-way ANOVA was performed followed by Holm-Sidak post-hoc test.
